# Hydroxychloroquine for post-exposure prophylaxis of COVID-19 among naval personnel in Sri Lanka: study protocol for a randomized, controlled trial

**DOI:** 10.1186/s13063-020-04659-7

**Published:** 2020-08-27

**Authors:** Madunil A. Niriella, Dileepa S. Ediriweera, Arjuna P. De Silva, Ranjan Premarathne, Priyantha Balasooriya, Kaluthanthri D. Duminda, Neelika G. Malavige, Kamani Wanigasuriya, Sarath Lekamwasam, Senanayake A. Kularathne, Sisira Siribaddana, Hithanadura J. de Silva, Saroj Jayasinghe

**Affiliations:** 1 Faculty of Medicine, University of Kelaniya, Ragama, Sri Lanka; 2 Navy Hospital, Welisara, Sri Lanka; 3 Army Hospital, Narahenpita, Sri Lanka; 4 Faculty of Medical Sciences, University of Sri Jayawardenapura, Nugegoda, Sri Lanka; 5 Faculty of Medicine, University of Ruhuna, Galle, Sri Lanka; 6 Faculty of Medicine, University of Peradeniya, Kandy, Sri Lanka; 7 Faculty of Medicine and Allied Sciences, Rajarata University of Sri Lanka, Anuradhapura, Sri Lanka; 8 Faculty of Medicine, University of Colombo, Colombo, Sri Lanka

**Keywords:** Randomized controlled trial, COVID-19, SARS-CoV-2, Prophylaxis, Post-exposure, Hydroxychloroquine, HCQ, Sri Lanka

## Abstract

**Background:**

The first case of a coronavirus 2019 (COVID-19) infection in a Sri Lankan was reported on March 11, 2020. The situation in Sri Lanka changed with the rapid increase of personnel contracting COVID-19 in a naval base camp that housed more than 4000 people. This provided a unique opportunity to study the effectiveness of hydroxychloroquine (HCQ) for post-exposure prophylaxis (PEP), while taking stringent, non-pharmacologic, public health measures to prevent spread. Our aim is to study the effectiveness and safety of HCQ for PEP among naval personnel with exposure to COVID-19-positive patients.

**Methods/design:**

This is a placebo-controlled, randomized, clinical trial carried out in the naval base camp and quarantine centers of the Sri Lanka Navy, Ministry of Defense, Sri Lanka. Navy personnel who are exposed to a patient with confirmed COVID-19 infection but test negative for the virus on reverse real-time polymerase chain reaction (rRT-PCR) at recruitment will be randomized, 200 to each arm, to receive HCQ or placebo and monitored for the development of symptoms or rRT-PCR positivity for severe acute respiratory syndrome coronavirus 2 (SARS-CoV-2) virus for 14 days.

**Discussion:**

This trial will provide high-quality evidence of the effectiveness and safety of HCQ as PEP for COVID-19. The study design is unique due to the circumstances of the outbreak in a confined area among otherwise healthy adults, at a relatively early stage of its spread.

**Trial registration:**

Sri Lanka Clinical Trials Registry (SLCTR) SLCTR/2020/011. Registered on 04 May 2020

## Background

The novel coronavirus 2019 (COVID-19) epidemic is a massive threat to public health worldwide. Current estimates suggest that the severe acute respiratory syndrome coronavirus 2 (SARS-CoV-2), responsible for COVID-19 illness, is both highly contagious (estimated basic reproductive rate, 2–3) and five- to fiftyfold more lethal than seasonal influenza (estimated mortality rate, 0.5–5%) [1]. Interventions to decrease the incidence and severity of COVID-19 are urgently needed.

The first case of COVID-19 in a Sri Lankan was reported on March 11, 2020, a tour guide who was exposed to European tourists. Since then, the cumulative numbers have increased. After a critical evaluation of evidence, the use of hydroxychloroquine (HCQ) in symptomatic patients with COVID-19 was included in a circular issued by the Ministry of Health, Sri Lanka, on March 27, 2020. Since then, the possible use of HCQ for pre- or post-exposure prophylaxis (PEP) has been intensely debated within the medical profession.

HCQ is an anti-malarial medication with immunomodulatory effects. HCQ has been shown to clear COVID-19 infection status and reduce viral shedding early during the disease course [2, 3]. Those against its introduction for prophylaxis cited the poor quality of the study designs and lack of reproducible results elsewhere. However, there were in vitro studies showing that it elevated endosomal pH, slowed the entry of COVID-19 into cells, and inhibited its replication [4, 5]. The drug is inexpensive, has a good safety profile—especially with short term use—and used extensively in the past to prevent malaria and currently used to treat autoimmune diseases [4, 6].

Clinical trials have been commenced in other countries to assess the efficacy of hydroxychloroquine as pre- or PEP to reduce the attack rate or severity among contacts of known or suspected COVID-19 patients [7–10]. India commenced pre-exposure prophylaxis of healthcare workers on March 22, 2020 [11]. In mid-April 2020, Indian scientists commenced mass prophylaxis of slum communities in Mumbai [12].

By late April, there were four studies from the USA, the UK, Singapore, and Canada to evaluate the role of HCQ in prophylaxis, registered in ClinicalTrials.gov [13–16].

The situation in Sri Lanka changed with the rapid increase of personnel contracting COVID-19 in a navy camp housing more than 4000 people in an area of about 52 acres. This was believed to have been the result of navy personnel being exposed to infected patients during contact tracing. This provided a unique opportunity to study the effectiveness and safety of HCQ in prophylaxis while taking stringent, non-pharmacologic, public health measures to prevent spread. Our research question is, “Is HCQ effective and safe for PEP among naval personnel with exposure to COVID-19-positive patients?”

## Methods

### Study design

This will be a 2-arm, placebo-controlled, randomized, clinical trial with a parallel group, 1:1 allocation ratio. The study will be carried out among all eligible persons meeting the inclusion/exclusion criteria at the naval base camp, Welisara, Sri Lanka, who are directed to be quarantined in quarantine centers (QCs) of the Sri Lanka Navy, Ministry of Defense, Sri Lanka. A patient information leaflet will be provided, and informed written consent will be obtained. Those willing to participate in the trial will be randomized and monitored for the development of symptoms or reverse real-time polymerase chain reaction (rRT-PCR) positivity for the SARS-CoV-2 virus. All eligible participants who test negative for the virus on rRT-PCR at baseline will be randomly assigned to the study arms within 48 h. Permission will be obtained through the navy to commence a double-blind randomized trial of HCQ to investigate its effectiveness and safety to prevent the development of COVID-19 infection in persons who are exposed but not yet infected.

### Study setting

The study settings are the naval base camp, Welisara, Sri Lanka, and the designated QCs maintained by the Sri Lanka Navy, Ministry of Defense, Sri Lanka.

### Study population

The study population is all the naval personal who are currently being directed for quarantine in QCs of the Sri Lanka Navy, Ministry of Defense, Sri Lanka.

#### Inclusion criteria

1. All consenting male and female adult naval personnel (18–59.9 years of age)
2. Exposure to a patient with confirmed COVID-19 infection (rRT-PCR positive for SARS-CoV-2 virus)
3. The exposure should be for > 30 min within 2 m of the infected individual and should be within 1 week at the time of inclusion in the study
4. Recruited within 48 h of admission to these centers
5. Willing to take study drug as directed for 5 days.

#### Exclusion criteria

1. Pregnant and lactating females
2. Suspected or confirmed to have COVID-19 infection (i.e., navy personnel having a positive rRT-PCR test for COVID-19 or the presence of fever with cough, shortness of breath, sore throat, anosmia, or diarrhea at the time of recruitment)
3. Presence of pre-existing cardiovascular disease including being on medication for ischemic heart disease
4. Presence of contraindication to the use of HCQ (on other medications predisposing to long QT interval or presence of long QT interval on baseline ECG)
5. Prior diagnosis of retinopathy, G6PD, malignancy, or advanced kidney disease
6. Known sensitivity or allergy to hydroxychloroquine

### Outcome measure

All participants will be monitored for the development of symptoms suggestive of COVID-19 and will undergo a rRT-PCR test for SARS-CoV-2 virus at the end of their quarantine period of 14 days.

#### Primary outcome measure

1. Number of participants testing positive for SARS-CoV-2 virus using rRT-PCR on day 14 at the completion of the quarantine period

#### Secondary outcome measures

1. Number of participants symptomatic for COVID-19 (symptomatic illness will be defined as the presence of fever with cough, shortness of breath, sore throat, anosmia or diarrhea from the time of recruitment until the completion of the quarantine period of 14 days)
2. Any reported adverse effects of HCQ

### Sample size

Altogether, 400 participants will be recruited to the study. Among them, 200 each will be allocated to the two arms: intervention and control arms.

### Sample size calculation

We used a simulation-based method to estimate the design power. The required sample size was determined by simulating 1000 samples for different sample sizes, and the minimum sample size which resulted in an 80% power to detect a significant intervention effect was selected. We considered the below assumptions to simulate data:

1. We assumed 50% of the study population have COVID-19 infections (i.e., but not diagnosed) at the time of recruitment.
2. If no intervention is adopted (i.e., control arm), we assumed 83.5% of the sample would be infected with COVID-19 infections (i.e., 66.7% increase) after 2 weeks.
3. If intervention is initiated, we expect a 25% reduction in the natural increase of COVID-19 infections after 2 weeks. Therefore, the intervention arm will experience 71.4% of COVID-19 infections.

Our simulation-based experiment showed the required minimal sample size as 190 for each arm to achieve 80% power. We considered additional 10% (i.e., 190/(1 − 0.1)) to allow loss to follow-ups and non-response bias. Therefore, the total required sample size for the study will be 211 persons for each arm.

### Consent, recruitment, and randomization

**Fig. 1:**
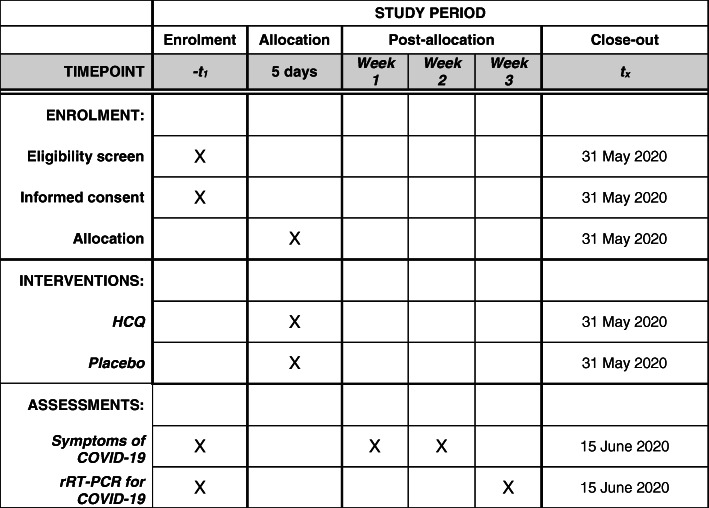
SPIRIT Figure for the trial - the schedule of enrolment, interventions, and assessments

Recruitment for the study will be performed in the main naval base camp in Welisara, Sri Lanka. The chief medical officer of the naval camp base, Welisara, Sri Lanka, is responsible for the briefing of the study details to potential participants, obtaining informed consent, and recruitment. Those who are willing to participate will be formally recruited after obtaining informed written consent (Fig. 1).

Permuted block randomization method will be used to allocate the participants to the intervention and placebo groups. Placebo will be produced similar to HCQ tablets in both size and shape. Allocation concealment will be maintained by packing the interventional product and the placebo in sequentially numbered, opaque, sealed envelopes with a similar appearance.

These envelopes will be numbered sequentially from 1 to 400 and will be handed over to the naval base camp in Welisara, Sri Lanka, without revealing which envelopes contain HCQ and placebo to ensure double blindness.

After randomization, study participants will be handed over the concealed intervention. Thereafter, the participants will be moved to the designated quarantine centers maintained by the Sri Lanka Navy. Once the participants reach the QC, they will be monitored for the continuation of intervention, compliance, and adverse effects by a designated chief nursing officer in each QC. There are 45 QCs maintained by the Sri Lanka Navy, Ministry of Defense, Sri Lanka, distributed throughout Sri Lanka.

- Arm 1: Intervention arm (*n* = 200): Participants will receive a loading dose of oral HCQ 400 mg (2 tablets) 12 hourly day 1 followed by oral HCQ 200 mg (1 tablet) 12 hourly for the next 4 days.
- Arm 2: Control arm (*n* = 200): Participants will receive an oral matching placebo (oral tablet containing 100 mg of elemental calcium) 2 tablets 12 hourly day 1, 12 hourly on day 1, followed by 1 tablet orally, 12 hourly for the next 4 days.

### Trial steering committee

The trial steering committee comprised several authors (MAN, JB, and HJdeS). This committee will be responsible for overseeing the recruitment and randomization as well as monitoring the adherence to the intervention, any reported adverse effects, and interim analysis. The trial steering committee (TSC) will also be responsible for the communication with the ERC. This committee will meet once a week to oversee the trial conduct and progress. An unblended statistician (author DSE) will audit the trial every 2 weeks and provide recruitment data to TSC.

The TSC is responsible for any protocol amendments and to notify the sponsor, ethics review committee, and the Sri Lanka Clinical Trial Registry. The PI will notify the quarantine centers regarding the amendments, and a copy of the revised protocol will be sent to the investigator site file. Any deviations from the protocol will be fully documented using a breach report form.

### Data collection method

Two trained doctors of the Sri Lanka Navy, in personal protective equipment, will obtain informed written consent from all the participants at the naval base camp, Welisara, Sri Lanka. Demographic details, co-morbidities, and exposure history will be collected via an interviewer-administered questionnaire at the recruitment. An ECG will be performed at the Navy Hospital, Welisara, Sri Lanka, on all participants to assess for pre-existing prolonged QT interval. A rRT-PCR for SARS-CoV-2 virus will be performed on all participants prior to recruitment (which was done as part of the existing screening program for COVID-19 of the Sri Lanka Navy) and after the completion of the quarantine period of 14 days. This sample collection for rRT-PCR will be performed by the already available, trained SL Navy personnel designated to perform this task, with the necessary precautions including personal protective equipment.

Samples from the nasopharynx will be obtained and transported in viral transport media and shipped at 4 °C to the laboratory at the Center for Dengue Research, Faculty of Medical Sciences, University of Sri Jayawardenapura, Sri Lanka, for analyses by one of the authors (NM) who will be blinded to the randomization. Confirmation of cases of COVID-19 will be based on the detection of unique nucleic acid sequences of virus RNA by rRT-PCR [17]. The collected biological material once used for the present study will not be stored or used for future any ancillary studies.

All enrolled participants will be monitored for possible adverse effects of the intervention and the placebo for the period of the study. In case of the development of symptoms suggestive of COVID-19 infection or any adverse events, a participant will be admitted to the Navy Base Hospital, Welisara, Sri Lanka.

### Data analysis

All main analyses will be based on an intention-to-treat principle. Baseline characteristics will be summarized by a treatment group to characterize the study sample and identify potential baseline imbalance. Post-interventional prevalence of SARS-CoV-2 virus for the control and intervention group will be calculated and presented. Subgroup analysis will be done for gender and age categories. A generalized linear mixed effect model will be used to evaluate the effectiveness of HCQ as PEP. A secondary analysis includes adjustment for potential confounders including body weight and metabolic co-morbidities (i.e., diabetes, hypertension, and dyslipidemia); multi-level models will address the dependence due to quarantine centers (i.e., random intercept will be introduced to for each QC). Additional analyses will include interactions to determine whether the effects differ by gender or age categories. Treatment effect will be presented using odds ratio with 95% confidence intervals, and *P* values for the treatment effect will be presented.

If the drop-out process is missing completely at random (MCAR), unbiased estimates of the intervention effects at post-randomization will be obtained. If missing data patterns are found to depend on specific baseline covariates, inverse probability-weighted generalized estimating equations (GEE) can be used to account for this in order to test the sensitivity of results to assumptions. If the models do not converge, multicollinearity of the variables and outliers will be addressed and refit the models and the number of iteration will be increased and reanalysis will be conducted.

R programming language version 3.6.3 will be used for the analyses.

### Ethical clearance

Ethical clearance for the study was obtained from the Ethics Review Committee (ERC) of the Faculty of Medicine, University of Kelaniya, Ragama—P/22/04/2020.

### Trial registration

This trial was registered in the Sri Lanka Clinical Trials Registry (SLCTR)—SLCTR/2020/011—on May 4, 2020 (https://slctr.lk/trials/slctr-2020-011), and authorization for the trial was obtained from the National Medicinal Regulatory Authority (NMRA)—CLITRI/2020/00032. WHO Universal Trial Number (UTN) for the study is U1111-1251-3613.

### Main ethical issues

All participants will be provided with a detailed information sheet in their preferred language. They will be allowed to provide informed written consent without undue influence. The collected biological material once used for the present study will not be stored or used for future any ancillary studies.

HCQ has been used for a long period of time for the treatment of a variety of illnesses, and its safety profile is well documented [6]. At the dosage given in this study, investigators do not envisage significant challenges with its safety. There is no anticipated harm and compensation for trial participation.

However, participants will be monitored daily, and all instances of adverse events will be documented. Furthermore, this trial will include generally fit participants (i.e., navy personnel on active duty) who have had medical examinations. All participants will have an ECG to exclude the pre-existing prolongation of the QT interval.

All serious adverse events and suspected unexpected serious adverse reactions (SUSARs) will be reported according to the guidelines issued by the National Medicines Regulatory Authority (NMRA), Sri Lanka, to the NMRA and ERC. Serious adverse events and SUSARs that require expedited reporting will be reported within stipulated timelines. All adverse events will be managed as appropriate by the trial physicians.

Trial progress will be monitored by an unblinded trial statistician. The trial will be terminated if there is an unexpectedly high serious adverse event rate among trial participants and/or evidence of futility. Further, unblinded data will be sent weekly to the Ethical Review Committee (which will in effect act as a data monitoring committee for this study).

### Budget

This clinical trial will be done in collaboration with the State Pharmaceutical Manufacturing Cooperation and Sri Lanka Navy, Ministry of Defense. All the necessary rRT-PCR test kits, drugs, and placebo for the study have been donated to the SL Navy. rRT-PCR testing will be done free of charge at the Department of Microbiology, Faculty of Medical Sciences, University of Sri Jayewardenepura, in accordance with the already existing protocol of the assessment of quarantined persons.

## Discussion

The study has a unique design due to the outbreak of COVID-19 infection in a confined area, among otherwise healthy adults at a relatively early stage of its spread. The space of 52 acres houses naval personnel in dormitory-type accommodation was ideal for rapid spread, and immediate measures were taken to send the initial positive cases to hospitals, transfer contacts to other QCs, and for segregation of personnel to minimize contact. Other preventive measures such as handwashing and physical distancing are also stringently followed. The communication of the completed trial results will be via a publication in a peer-reviewed, high-impact journal.

### Trial status

Trial protocol version 2.0 dated May 02, 2020, was granted ethical approval and regulatory registration. Recruitment began on May 04, 2020, and the approximate date on which recruitment will be completed will be May 31, 2020.

## Data Availability

Individual participant data which is used to generate results will be shared immediately following publication after de-identification (text, tables, figures, and appendices). The study protocol, statistical analysis plan, and analytic code will be made available. Data will be shared with investigators whose proposed use of the data has been approved by an independent review committee identified for this purpose. Data will be shared to achieve the aims in an approved proposal. Proposals should be directed to maduniln@yahoo.co.uk. To gain access, data requestors will need to sign a data access agreement. Similarly, the participant information sheet, consent form, and data collection form are available from the corresponding author (MAN) on request.

## Abbreviations

SARS-CoV-2: Severe acute respiratory syndrome coronavirus 2
COVID-19: Novel coronavirus infection 2019
HCQ: Hydroxychloroquine
PEP: Post-exposure prophylaxis
QCs: Quarantine centers
rRT-PCR: Reverse real-time polymerase chain reaction
ERC: Ethics Review Committee
SLCTR: Sri Lanka Clinical Trials Registry
NMRA: National Medicinal Regulatory Authority
